# A Hydrogel Model Incorporating 3D-Plotted Hydroxyapatite for Osteochondral Tissue Engineering

**DOI:** 10.3390/ma9040285

**Published:** 2016-04-14

**Authors:** Michal Bartnikowski, Ashwini Rahul Akkineni, Michael Gelinsky, Maria A. Woodruff, Travis J. Klein

**Affiliations:** 1 Institute of Health and Biomedical Innovation, Queensland University of Technology, 60 Musk Avenue, Kelvin Grove, Queensland 4059, Australia; m.bartnikowski@qut.edu.au; 2 Centre for Translational Bone, Joint and Soft Tissue Research, Faculty of Medicine, Technische Universität Dresden, Fetscherstraße 74, Dresden D-01307, Germany; Ashwini_Rahul.Akkineni@tu-dresden.de (A.R.A.); michael.gelinsky@tu-dresden.de (M.G.)

**Keywords:** cartilage tissue engineering, hydrogel, 3D plotting, hydroxyapatite, gelatin, alginate, chondrocyte, chondrogenesis

## Abstract

The concept of biphasic or multi-layered compound scaffolds has been explored within numerous studies in the context of cartilage and osteochondral regeneration. To date, no system has been identified that stands out in terms of superior chondrogenesis, osteogenesis or the formation of a zone of calcified cartilage (ZCC). Herein we present a 3D plotted scaffold, comprising an alginate and hydroxyapatite paste, cast within a photocrosslinkable hydrogel made of gelatin methacrylamide (GelMA), or GelMA with hyaluronic acid methacrylate (HAMA). We hypothesized that this combination of 3D plotting and hydrogel crosslinking would form a high fidelity, cell supporting structure that would allow localization of hydroxyapatite to the deepest regions of the structure whilst taking advantage of hydrogel photocrosslinking. We assessed this preliminary design in terms of chondrogenesis in culture with human articular chondrocytes, and verified whether the inclusion of hydroxyapatite in the form presented had any influence on the formation of the ZCC. Whilst the inclusion of HAMA resulted in a better chondrogenic outcome, the effect of HAP was limited. We overall demonstrated that formation of such compound structures is possible, providing a foundation for future work. The development of cohesive biphasic systems is highly relevant for current and future cartilage tissue engineering.

## 1. Introduction

Articular cartilage is a complex tissue, organized into a zonal structure with varied biochemical and biomechanical properties throughout each zone. Cartilage matrix is rich in collagens and proteoglycans, with the dominant collagen type (>90%) being collagen type II [1]. The zones of cartilage vary with depth, with collagen fiber orientation and chondrocyte morphology changing throughout the tissue, from the superficial layer (fibers parallel to surface, flattened cell morphology), to the transitional layer (fibers randomly oriented, rounded cell morphology) and finally the deep layer (fibers perpendicular to surface, columnar cell alignment). The zone of calcified cartilage (ZCC) is found beyond the deepest part of cartilage, linking the cartilage to the subchondral bone. The ZCC varies considerably from the cartilage, for instance in collagen II content: ~60% dry weight in cartilage *versus* ~20% in the ZCC [2]. Further, the percentage of hydroxyapatite (HAP) in the ZCC by dry weight is ~65%, comparable with that of subchondral bone at ~86% [2]. The ZCC is also rich in collagen X, which is absent from hyaline cartilage [3]. It is overall a transitional tissue, providing a gradient of mechanical properties between the more compliant articular cartilage and the stiffer underlying bone tissue [3].

As intrinsic cartilage repair is poor, numerous strategies for regeneration have emerged, such as the tissue engineering of cartilage scaffolds [4,5,6,7]. This approach predominantly revolves around the use of hydrogels, highly hydrated and diffusive polymer matrices which provide nutrient exchange and a three-dimensional (3D) matrix whilst also providing a desired chondromimetic hypoxic environment [8,9]. 3D-printing/plotting has also been used in tissue engineering of cartilage or osteochondral defects, wherein deposition of a polymer (melt or hydrogel) [10,11] and/or ceramic paste (in the case of osteochondral scaffolds) [12,13] is used to form a mesh-type structure on a computer-controlled stage, resulting in a scaffold structure with a desired morphology. Whilst hydrogel matrices are highly suited to work towards cartilage regeneration, bridging the gap into engineering full osteochondral defects is difficult and more complex [12].

Herein we focus on the development of a novel scaffold system constructed using a combination of 3D plotting and gel casting with photopolymerization. Gelatin, commonly produced through hydrolysis of extracellular matrix (ECM) collagens such as those found in bone and skin, retains desired matrix metalloproteinase (MMP)-sensitive degradation sites and cell binding motifs such as Arg-Gly-Asp (RGD) [14]. It is also chemically modifiable, yielding a photocrosslinkable hydrogel gelatin methacrylamide (GelMA) [15]. Hyaluronic acid (HA), the most abundant glycosaminoglycan (GAG) found in cartilage, is also similarly modifiable (hyaluronic acid methacrylate, HAMA) and has been shown to promote chondrogenic differentiation *in vitro* [16]. In this work, we print grid scaffolds made of an alginate/HAP paste to form a ZCC and combine these with a GelMA ± HAMA hydrogel system through ultraviolet (UV) photopolymerization. We hypothesize that such constructs will ultimately be beneficial in innovation towards a complete osteochondral scaffold by entrapping hydroxyapatite in proximity to chondrocytes and hence potentiating the development of a ZCC.

## 2. Experimental Section

Gelatin (G2500; ~300 g bloom), and methacrylic anhydride (MAAh, 276685) were purchased from Sigma (Sigma-Aldrich, St. Louis, MO, USA). Hyaluronic acid (HA; molecular weight (MW) 0.86 MDa) was purchased from Novozymes. 2-hydroxy-1-[4-(2-hydroxyethoxy)phenyl]-2-methyl-1-propanone (Irgacure 2959; IC) was purchased from BASF (BASF, Ludwigshafen, Germany). All quoted concentrations in percent are weight per volume (% *w*/*v*) unless otherwise noted. Alginate (9005-38-3) and methyl cellulose (9004-67-5 M0512, MW 88 kDa) were purchased from Sigma (Sigma-Aldrich, Steinheim, Germany) and hydroxyapatite (1.02196.1000) was purchased from Merck (Darmstadt, Germany). Hydroxyapatite particle size was previously quantified using Transmission Electron Microscopy (TEM; F30, FEI, Zürich, Switzerland, operated at 200 kV), and found to comprise needle-shaped particles with a length of 50 ± 4 nm and width of 17 ± 2 nm [17]. Calcium chloride dihydrate (10035-04-8) was purchased from Roth (Karlsruhe, Germany).

### 2.1. Polymer Functionalization

Functionalization of gelatin (to gelatin methacrylamide, GelMA) and HA (to hyaluronic acid methacrylate, HAMA), was performed as previously described [18,19]. Briefly, gelatin and HA were dissolved in Milli-Q water (Merck Millipore, Billerica, MA, USA) at 10% and 1%, respectively. In the case of gelatin, 0.6 g of MAAh per 1 g was added and reacted for 1 h at 50 °C [18]. HA was reacted with a 5-fold molar excess of MAAh to hydroxyl groups for 8 h on ice, with pH monitored and adjusted to 8 with 5 M NaOH [19]. After reaction, all gel solutions were subject to centrifugation to phase separate and allow the removal of insoluble MAAh. HAMA was precipitated with ice-cold 100% ethanol and collected, after which both were separately dialyzed against Milli-Q water to allow the diffusion of any remainder of MAAh or methacrylic acid. After dialysis, GelMA was filtered through 0.2 µm filters (Merck Millipore) under aseptic conditions, pH adjusted to 7.4, lyophilized with 0.2 µm filters exclusively providing air exchange then stored at −20 °C in sealed, sterile tubes. HAMA was subject to the same processing apart from initial filtration to avoid macromer loss due to the large molecular weight.

### 2.2. Degree of Functionalization

The degree of functionalization (DOF) of GelMA and HAMA was assessed using proton nuclear magnetic resonance (^1^H NMR) spectroscopy. A Varian Direct Drive NMR spectrometer (Agilent Technologies, Santa Clara, CA, USA) operating at 400 MHz for hydrogen, was used to record ^1^H NMR spectra. All samples were dissolved at 1.0% in D_2_O at a temperature of 50 °C. Spectra were recorded at an operational temperature of 50 °C, with 32 scans and a recycle delay of 30 s. For GelMA, methacrylamide shifts were normalized against the aromatic signal of phenylalanine, at δ 7.4 ppm [20]. DOF was defined as the proportion of modified lysine groups of gelatin, as previously described for collagen I methacrylamide [20]. The area obtained from the integral of intensities of the protons present at the methacrylamide carbon-carbon double bond ($I_{DB}$) occurring at δ 5.6 and 5.8 ppm was normalized to proton number ($n_{H_{DB}}$), and the area obtained from integrating the peak of the aromatic groups ($I_{A}$) was normalized to the protons interacting with the aromatic ring ($n_{H_{A}}$). The prevalence of modified groups and aromatic residues were then normalized to their prevalence by number in porcine gelatin in order to allow for the quantitation of total methacrylamide to total possible lysine groups (Equation (1)) [21]:

(1) $$DOF_{GelMA}\;\left(\%\right)=\;\frac{\left(\frac{I_{DB}}{n_{H_{DB}}}\right)/\%\;Lys}{\left(\frac{I_{A}}{n_{H_{A}}}\right)/\%\;Phe}$$

For HAMA, chemical shifts were normalized against the signal from the n-acetyl group on the HA backbone at δ 2.1 ppm [22]. The DOF, which in this case was defined as the fraction of modified hydroxyl groups per repeating unit, was determined as previously described [22]. In brief, the average area found from integrating the proton peak at the double bond (~δ 5.8 and 6.3 ppm; $I_{DB}$) was normalized to the number of protons at the double bond ($n_{H_{DB}}$) and the area found from integrating the proton peak of the n-acetyl group ($I_{CH_{3}}$) was normalized to the number of protons in the n-acetyl group ($n_{H_{CH_{3}R}}$); the normalized double bond value was then divided by the normalized n-acetyl group value to give the number of functional groups, which was assessed as a fraction of $n_{OH_{M}}$, the total number of target reactive hydroxyl groups (4) present in a repeating monomer unit of HA, finally resulting in the percentage of DOF, as illustrated below (Equation (2)):

(2) $$DOF_{HAMA}\;\left(\%\right)=\;\frac{\frac{I_{DB}}{n_{H_{DB}}}/\frac{I_{CH_{3}R}}{n_{H_{CH_{3}R}}}}{n_{OH_{M}}}\times 100$$

### 2.3. Hydroxyapatite/Alginate Scaffold Plotting

Pastes were made containing 42% (*wt*/*wt*) HAP, 1.74% (*wt*/*wt*) alginate and Milli-Q H_2_O (for ALG/HAP scaffolds), and 10% (*wt*/*wt*) methyl cellulose, 1.74% (*wt*/*wt*) alginate and Milli-Q H_2_O (for pure alginate (ALG) control scaffolds). An iterative process was used for the development of the final paste composition, with the above blend of alginate and methyl cellulose providing highest fidelity and structural integrity. Pastes were 3D plotted in air using a BioScaffolder 2.1 (GeSiM, Großerkmannsdorf, Germany) into a 4 × 4 × ~0.5 mm grid using a 410 µm (inner diameter) needle, with a printing speed of 3 mm·s^−1^. Extrusion pressure of 380 kPa and 250 kPa were used for ALG/HAP and pure ALG scaffolds, respectively. A start-break (the application of pressure in advance of the printing of a strand, *i.e.*, during the approach of the nozzle to the designated start point) of 0.5 s was used to ensure the initial region of each strand was printed with a stabilized flow. Further information regarding printing parameters may be found in Kumar *et al.* [17]. Scaffold design consisted of 2 printed layers with 4 strands per layer. After plotting, scaffolds were stored in 1 m CaCl_2_ solution at 4 °C until the day of cell encapsulation.

### 2.4. Micro-Computed Tomography Imaging

Micro-Computed Tomography (µCT) was used to qualitatively assess constructs containing ALG and HAP (Scanco Medical µCT 40, operated at 55 kVp and 145 µA; Scanco Medical, Brüttisellen, Switzerland), and determine HAP localization within the constructs.

### 2.5. Cell Isolation and Expansion

The full procedure for isolating chondrocytes is described elsewhere [23]. In brief, cartilage samples were removed from macroscopically normal regions of the femoral condyle of a patient undergoing full knee replacement surgery, with patient consent and ethical approval from the Prince Charles Hospital and Queensland University of Technology (Ethics RM: 1400001024). After excision, chondrocytes were cultured and expanded in low-glucose DMEM with 10% fetal bovine serum (Lonza, Waverly, Australia), 2 mM glutamax, 10 mM 4-(2-hydroxyethyl)-1-piperazineethanesulfonic acid (HEPES), 0.1 mM non-essential amino acids (NEAAs), 0.5 µg·mL^−1^ amphotericin B (Fungizone^®^; Thermo Fisher Scientific, Waltham, MA, USA), 50 U·mL^−1^ penicillin G sodium, 50 µg·mL^−1^ streptomycin (all Invitrogen; Thermo Fisher Scientific, Waltham, MA, USA), 0.4 mM l-proline and 0.1 mM ascorbic acid (Sigma) and used at passage 3.

### 2.6. Hydrogel Formation and Culture

**Figure 1:**
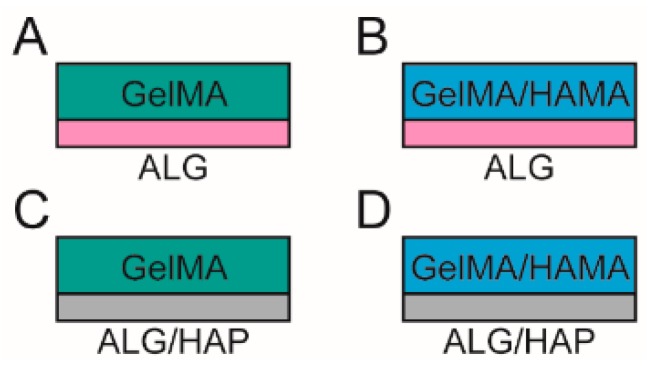
Schematic illustration of the groups included in the study.Groups used in the study comprised (**A**): gelatin methacrylamide (GelMA) with an alginate (ALG) paste scaffold; (**B**): GelMA with hyaluronic acid methacrylate (HAMA) with an ALG paste scaffold; (**C**): GelMA with a combined alginate and hydroxyapatite paste (ALG/HAP) scaffold; and (**D**): GelMA/HAMA with an ALG/HAP scaffold.

Stock solutions of GelMA, HAMA and IC were made in PBS on the day of gel formation. Groups consisted of (Figure 1): 10% GelMA/ALG control scaffold (GelMA-ALG); 10% GelMA/0.5% HAMA/ALG control scaffold (GelMA/HAMA-ALG); 10% GelMA/HAP scaffold (GelMA-ALG/HAP); and 10% GelMA/0.5% HAMA/HAP scaffold (GelMA/HAMA-ALG/HAP).

ALG and ALG/HAP 3D plotted scaffolds were placed into custom Teflon molds (50 × 4 × 2 mm strips) and rinsed twice with DMEM to reduce calcium content from storage. Hydrogel precursor solutions containing 0.05% IC were equilibrated at 37 °C for 15 min in an incubator. Chondrocytes at a density of 10^6^ mL^−1^ were added, and the mixtures were pipetted into the molds over the scaffolds and covered with glass slides. Storage of 10 min at 4 °C was performed to allow GelMA to physically crosslink, equilibrate groups, and minimize cell settling. UV crosslinking was done using 365 nm light for a total energy of 1500 mJ·cm^−2^ (2.3 mW·cm^−2^ for 11 min) in a CL-1000 L crosslinker (UVP, Upland, CA, USA). Cell-free groups were made in the same fashion, with cellular volume accounted for in dilutions. After crosslinking, gel strips were cut to 4 × 4 × 2 mm and divided into 48-well plates. After gels were divided, they were cultured for 28 days in chondrogenic differentiation media (high-glucose DMEM with 2 mM glutamax, 10 mM HEPES, 0.1 mM NEAAs, 0.5 µg·mL^−1^ amphotericin B (Fungizone^®^; Thermo Fisher Scientific: Waltham, MA, USA), 50 U·mL^−1^ penicillin G sodium, 50 µg·mL^−1^ streptomycin, ITS-G (100× dilution; all Life Technologies), 1.25 mg·mL^−1^ bovine serum albumin (BSA), 0.4 mM l-proline, 0.1 mM ascorbic acid, 0.1 µM dexamethasone (all Sigma) and 10 ng·mL^−1^ transforming growth factor-β3 (TGF-β3; GroPep, Adelaide, Australia). Media was changed three times a week.

### 2.7. Viability Assay

Viability was assessed on day 1 of culture (*n* = 3 samples per group). Gels were cut in half and thereafter treated with fluorescein diacetate (FDA) and propidium iodide (PrI; both Invitrogen; Thermo Fisher Scientific: Waltham, MA, USA) to stain live and dead cells, respectively. Gels were rinsed twice in fresh PBS then incubated for 5 min at 37 °C in a 5 µg·mL^−1^ FDA, 0.5 µg·mL^−1^ PrI PBS solution. Samples were imaged across the cross-section using fluorescence microscopy (Zeiss Axio Imager M2; excitation: 488 nm and 568 nm, absorption 520 nm and 640 nm for FDA and PrI, respectively—1.33 µm·pixel^−1^). Quantification was performed using ImageJ software (National Institutes of Health, New York, NY, USA). As chondrocytes were used in this study, culture plate positive and negative staining controls were not used—the cells would be unable to recover a rounded morphology in this setting and thus provide erroneous control data.

### 2.8. Mechanical Properties

Mechanical testing was performed on days 1 and 28 on cell-free and cell-containing samples using an Instron MicroTester (Model 5848; Instron, Norwood, MA, USA). Each sample was placed in a 37 °C PBS trough into which an aluminum plunger was submerged for unconfined compressive displacement. The testing regimen consisted of a preliminary height measurement (Δforce (F) = −0.01 N as height criterion), followed by a 0.5% strain (ε)/s (s) ramp to 16% ε for compressive elastic modulus measurement. The value for elastic modulus was derived from the slope of the stress-strain curve from 10% to 15% strain.

### 2.9. Biochemical Assays

Gels harvested on days 1 and 28 were disrupted by passing through a 19 G needle and digested in 0.5 mg·mL^−1^ Proteinase K solution, on a shaker plate overnight at 56 °C. The DNA content was quantified using the Quant-iT™ PicoGreen^®^ dsDNA assay kit (Invitrogen; Thermo Fisher Scientific: Waltham, MA, USA) according to the manufacturer’s instructions.

GAG content of proteinase K-digested hydrogel and culture media samples were assessed using the dimethyl-methylene blue (DMMB) assay (pH 1.5) (sample GAG on days 1 and 28, media on days 14 and 28). Absorbances at 525 and 590 nm were taken, with the ratio of the two absorbances normalized to a chondroitin sulfate C (Sigma) standard to give the GAG content. All GAG content measurements were normalized using cell-free constructs to account for GAGs released from the hydrogels *versus* GAGs produced by the cells.

### 2.10. Gene Expression

RNA was extracted from constructs cultured for 28 days; gels were frozen at −80 °C in 1 mL of TRIzol reagent (Invitrogen; Thermo Fisher Scientific: Waltham, MA, USA), and RNA was extracted according to manufacturer’s instructions. SuperScript™ III First Strand Synthesis System (Invitrogen; Thermo Fisher Scientific, Waltham, MA, USA) was used to synthesize complementary DNA (cDNA). DNase digestion was performed prior to cDNA synthesis and RNase digestion was performed after.

Primer sequences were either obtained from the literature for *RPL13A*, *COL1A1*, *COL2A1*, *ACAN* and *COL10A1* [9,24] or designed using Primer-BLAST (NCBI, Bethesda, MD) for *TBP* (5′→3′ F:AGCCAAGAGTGAAGAACAGTC, R:CATCACAGCTCCCCACCATATT). Real-time quantitative reverse transcriptase polymerase chain reactions (PCR) were performed in duplicates in a 7500 PCR system (Applied Biosystems, Foster City, CA, USA) using SybrGreen Mastermix (Applied Biosystems/Invitrogen; Thermo Fisher Scientific: Waltham, MA, USA). The cycle threshold (Ct) value of each gene was normalized to the geometric mean of the housekeeping genes *RPL13A* and *TBP* using the comparative Ct method (2^−∆Ct^).

### 2.11. Immunofluorescence

Gels from day 28 were frozen in Optimal Cutting Temperature compound (OCT, Sakura, Finetek, Tokyo, Japan) and sectioned on a cryo-microtome at −20 °C. Sections were fixed with ice-cold acetone for 15 min, followed by antigen retrieval with 0.1% hyaluronidase (Sigma) for 30 min, which was only included in the collagen I and II staining procedures. Primary antibodies were diluted in 2% goat serum in PBS (Jackson ImmunoResearch, West Grove, PA, USA), and applied overnight at 4 °C in a moist chamber. Antibodies for collagen type I (I-8H5, MP Biomedicals, Seven Hills, Australia, 1:300 dilution), collagen type II (II-II6B3, Developmental Studies Hybridoma Bank (DSHB), Iowa City, IA, USA, 1:200 dilution), collagen type X (14-9771-82 Clone X53, Jomar Bioscience Pty Ltd., Kensington, Australia, 1:300 dilution), aggrecan (969D4D11, Life Technologies 1:300 dilution) or isotype controls (mouse IgG, Jackson, 1:1000) were used. Subsequently, sections were incubated in 2% donkey serum/PBS with IgG_1_-specific AlexaFluor^®^488-labelled goat anti-mouse secondary antibodies and IgG_2a_-specific AlexaFluor^®^594-labelled goat anti-mouse secondary antibodies (Jackson 1:150) and 5 µg·mL^−1^ 4′,6-diamidino-2-phenylindole (DAPI, Sigma) for 1 h at room temperature. Images were captured with fixed exposure times on a Zeiss Axio Imager M2 microscope with epi-fluorescence attachment (Carl Zeiss, Jena, Germany).

### 2.12. Statistical Analyses

Analysis of Variance (ANOVA) tests were used to assess differences between groups, with Tukey’s post-hoc tests used to determine inter-type relationships in groups with equal variances assumed (e.g., cell-free constructs); Dunnet’s T3 post-hoc test was used for cell-containing samples, assuming unequal variances. Tests were conducted using SPSS 21.0 (IBM Corporation: Armonk, NY, USA) with *p*-values < 0.05 regarded as significant. Figure bars show mean ± standard deviation. Captions state where independent sample *t*-tests were used instead of ANOVA. Statistically significant differences are indicated using Roman numerals or symbols; any groups sharing a Roman numeral are statistically similar whilst groups without a like numeral are different.

## 3. Results

### 3.1. Scaffold Manufacturing by 3D Plotting

**Figure 2:**
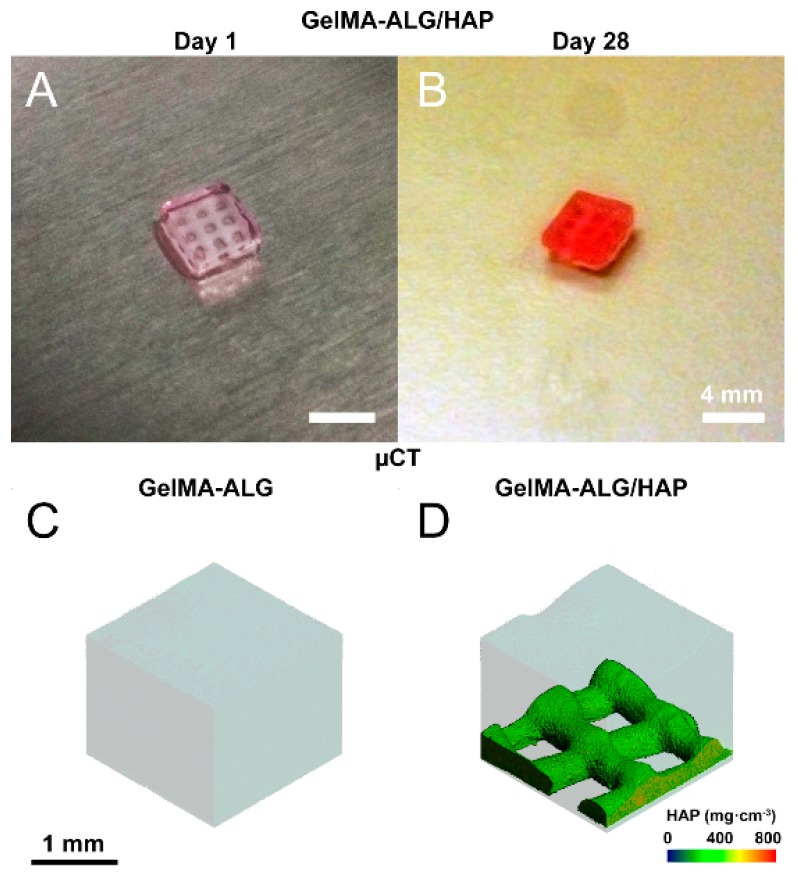
Images of hydrogel/scaffold hybrid constructs. (**A**) photo illustrating ALG/HAP scaffolds crosslinked within GelMA hydrogels on day 1 and (**B**) day 28. Scale bar 4 mm; (**C**,**D**) scaffolds imaged using micro-computed tomography (µCT), with thresholding used to isolate high density components (colored scale illustrates mg·cm^−3^ of HAP); (**C**) GelMA-ALG and (**D**) GelMA-ALG/HAP illustrated, scale bar 1 mm as indicated.

Scaffolds were successfully fabricated using the BioScaffolder 2.1 and remained intact from day 1 (Figure 2A) to day 28 of culture (Figure 2B). Visualization of scaffolds in combination with gels was done using µCT and confirmed localization of hydroxyapatite to the deep region of the construct (GelMA-ALG Figure 2C, GelMA-HAP Figure 2D). Four groups were chosen for the study (Figure 1), comprising HAP-containing (GelMA-ALG/HAP, GelMA/HAMA-ALG/HAP) and HAP-free (GelMA-ALG, GelMA/HAMA-ALG) constructs.

### 3.2. Degree of Functionalization

**Figure 3:**
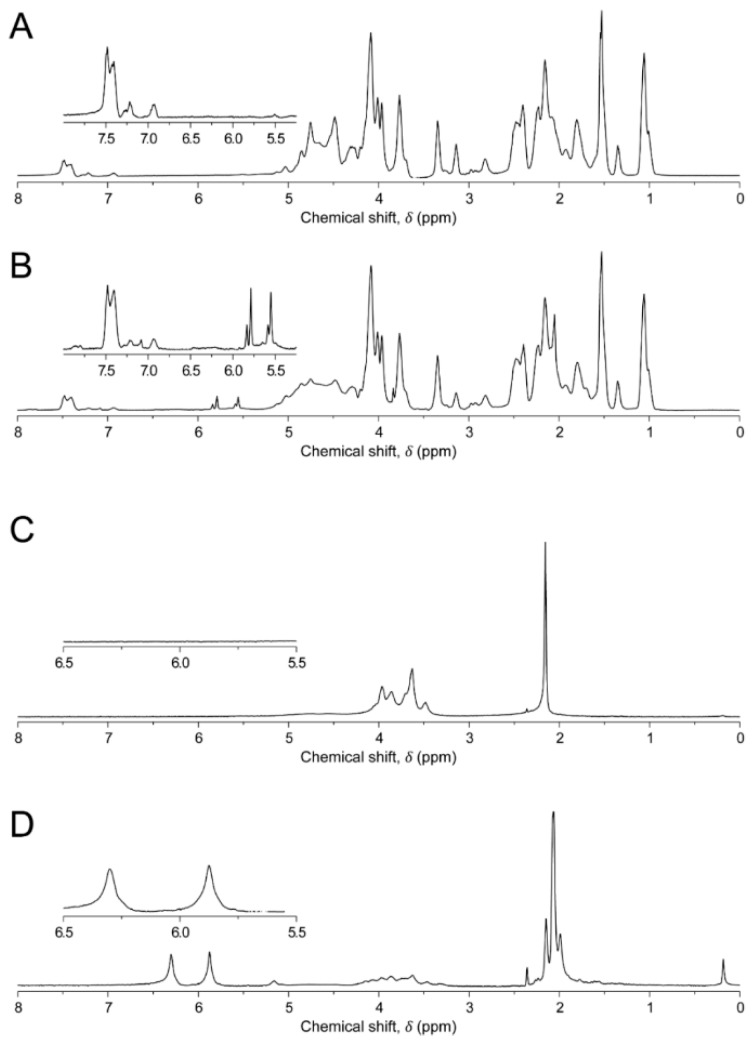
Proton Nuclear Magnetic Resonance (^1^H NMR) spectra of hydrogels. (**A**): gelatin; (**B**): gelatin methacrylamide (GelMA); (**C**): hyaluronic acid (HA); (**D**): hyaluronic acid methacrylate (HAMA). Areas of interest are inset within each frame. Aromatic peaks in gelatin/GelMA are present at ~chemical shift (δ) 7.4 ppm, with the two free protons on the methacrylate groups present at δ 5.6 and δ 5.8 ppm respectively. In HA, the peak from the n-acetyl protons is present at ~δ 2.1 ppm, with the methacrylate protons used for degree of functionalization (DOF) measurements present in HAMA at δ 5.8 and δ 6.3 ppm.

To form photocrosslinkable polymers, gelatin and hyaluronic acid were functionalized using varied molar excesses of MAAh. ^1^H NMR analysis was performed to obtain DOF from NMR spectra (Figure 3). GelMA was found to be 76.2% functionalized, and HAMA was 25.0%.

### 3.3. Viability

**Figure 4:**
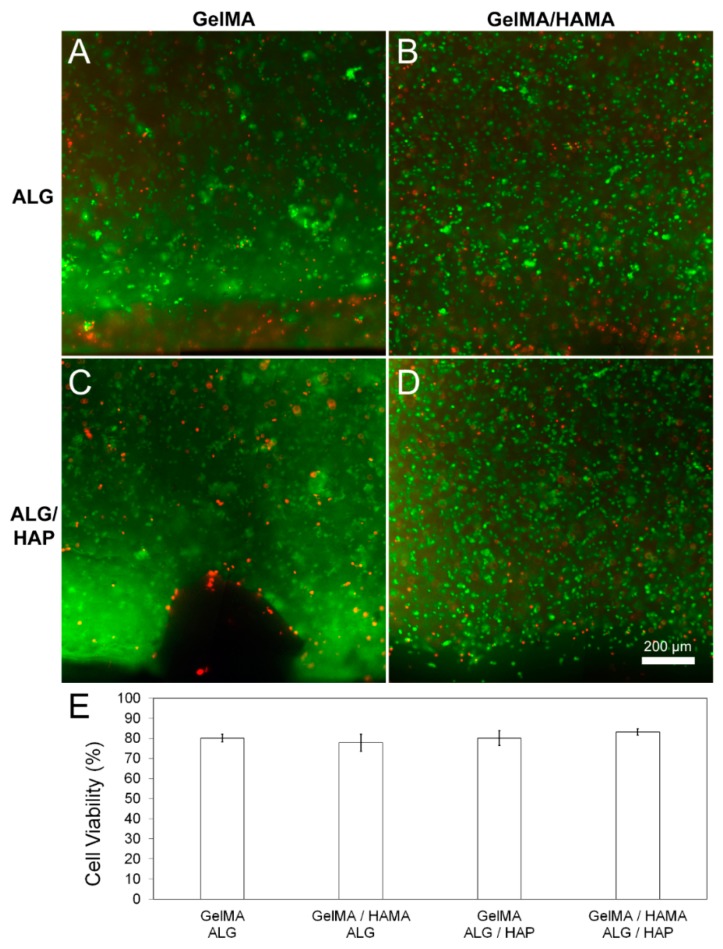
Cell viability in hydrogel groups on day 1 of culture. Images and quantitated data of cell viability measured using FDA/PI assay on day 1 of culture. Quantification and image analysis was performed using ImageJ. (**A**): GelMA-ALG; (**B**): GelMA/HAMA-ALG; (**C**): GelMA/ALG-HAP; (**D**): GelMA-HAMA/ALG-HAP; (**E**): quantified viability from *n* = 3 samples per group. Scale bar 200 µm.

Live/dead staining was performed using FDA (live) and PrI (dead) to assess cytotoxicity of the scaffolds or the hydrogel system. No abnormal cytotoxic effects were detected from scaffolds on day 1 of culture in any group (Figure 4A–D), with no significant difference between quantified scaffold viability (Figure 4E; *n* = 3 samples per group). A viability of 80% is an expected value considering chondrocyte expansion, encapsulation and processing through UV radical polymerization.

### 3.4. Mechanical Properties

**Figure 5:**
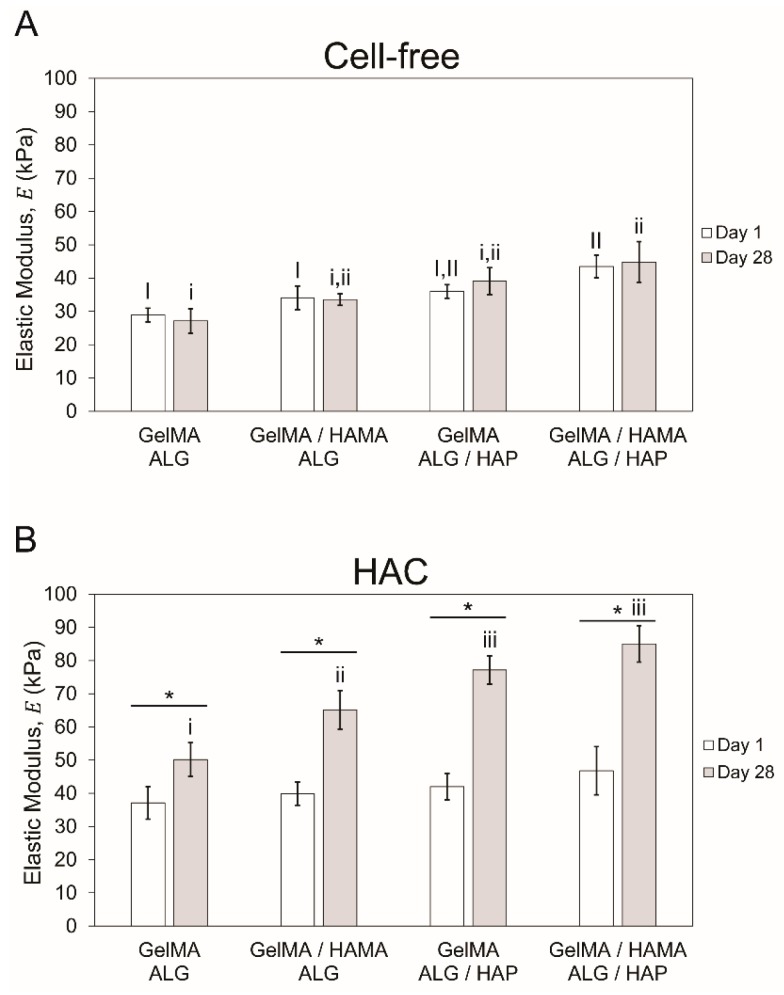
Mechanical testing results showing compressive elastic modulus. Elastic modulus of samples on days 1 and 28 of culture (*n* = 3 per group) with (**A**): cell-free and (**B**): cell-containing gels shown separately. The four groups in each time point were compared separately to the second time point, with upper case Roman numerals used for day 1 and lower case for day 28. When groups share Roman numerals they are statistically similar. Bars with stars indicate *t*-test comparison results between one group across two time points.

Mechanical testing was performed on cell-free and cell-containing (human articular chondrocytes, HAC) constructs to assess purely material-based stiffness as well as stiffness due to ECM deposition throughout culture. Elastic modulus was found to be slightly higher in cell-free HAP groups (Figure 5A; *n* = 3 per group), with significant difference only observed between GelMA-ALG, GelMA/HAMA-ALG and GelMA/HAMA-ALG/HAP on day 1. On day 28, difference was noted between GelMA-ALG and GelMA/HAMA-ALG/HAP. Modulus of cell-free constructs did not increase during culture, which ensured changes in cell-containing gels were cell-mediated. Cell-containing gels (Figure 5B; *n* = 3 per group) were not significantly different on day 1, but all significantly increased over the culture period. It was found that GelMA-ALG (~50 kPa) was significantly lower than GelMA/HAMA-ALG (~65 kPa), with the two HAP groups significantly higher than the ALG control groups but statistically similar to each other (GelMA/ALG-HAP: ~77 kPa, GelMA-HAMA/ALG-HAP: ~85 kPa).

### 3.5. DNA and GAG Content

**Figure 6:**
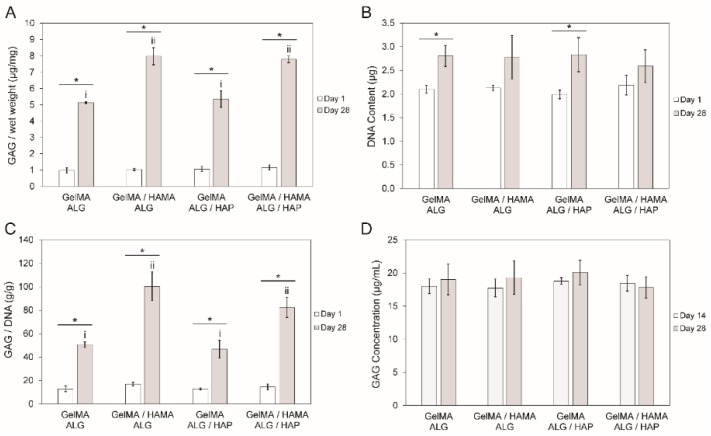
DNA and GAG content in constructs over the culture period. (**A**): comparison of day 1 and day 28 results of GAG content (µg) per gel wet weight (mg); (**B**): DNA content (µg) per gel; (**C**): GAG content (g) per DNA content (g) in gels and (**D**): GAG concentration in media (µg·mL^−1^) on days 14 and 28 of culture. The four groups in each time point were compared separately to the second time point, with upper case Roman numerals used for day 1 and lower case for day 28. When groups share Roman numerals they are statistically similar. Bars with stars indicate *t*-test comparison results between one group across two time points. All groups have *n* = 3 samples. In all cases cell-free constructs were used to correct for any material-based content potentially interfering with the GAG measurements.

Measurements of DNA content and GAG content were performed on all construct groups, with GAG content in media also measured. In all cases cell-free constructs were used to correct for any material-based content potentially interfering with the GAG measurements. These data were collected to illustrate whether there were proliferative differences between groups (DNA content), and to assess chondrogenesis (GAG content). Normalization of GAG content to DNA content was done to account for cell number skewing raw GAG content per wet weight data. Large increases in GAG content per wet weight were observed between days 1 and 28, with all groups increasing from between 5- and 8-fold (Figure 6A). GAG content per wet weight in the GelMA-ALG and GelMA-ALG/HAP groups were significantly different to the GelMA/HAMA-ALG and GelMA/HAMA-ALG/HAP groups, however no differences were found between ALG and ALG/HAP within each gel blend. Overall, HAMA appeared to have a positive effect on chondrogenesis during culture. The DNA content in constructs increased slightly over the culture period, which showed that cellular activity was maintained (Figure 6B). GAG content per DNA content data shared the same trends as GAG content per wet weight, as would be expected with stable DNA levels throughout the culture and between groups (Figure 6C). GAG content per media was found to be consistent between day 14 and day 28, indicating that a similar low level of GAG was lost to the media throughout culture (Figure 6D). Sample size of *n* = 3 per group was used in all cases.

### 3.6. Gene Expression

**Figure 7:**
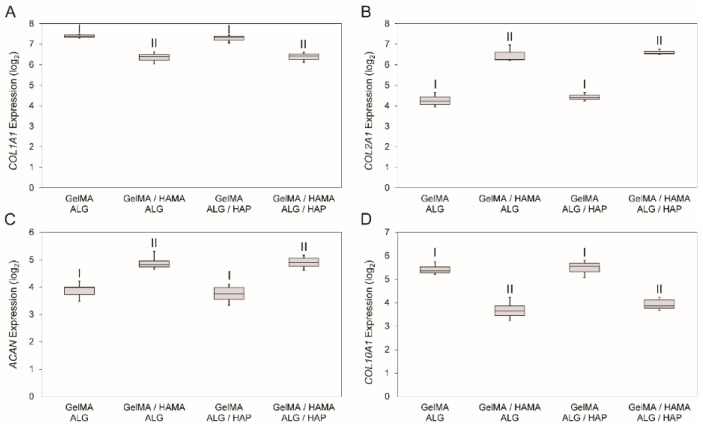
Gene expression data for the four groups from day 28 samples. The four genes that were analyzed were (**A**): *COL1A1*; (**B**): *COL2A1*; (**C**): *ACAN* and (**D**): *COL10A1* on day 28 of cell culture shown as 2^−ΔCt^ value (after log_2_ conversion). Statistical analysis was performed on log_2_ data with each group containing *n* = 3 samples. A Dunnet’s T3 post-hoc test was used to compare means, with *p* < 0.05 taken as significant. Shared Roman numerals indicate statistical similarity between gel groups.

Analysis of gene expression was performed to test the effects of materials on gene expression in the system. *COL2A1* and *ACAN* levels were tested to assess positive chondrogenesis, with *COL1A1* and *COL10A1* serving as indicators for fibrocartilage development and chondrocyte hypertrophy/calcification. Gene expression results showed an influence of HAMA, with a decrease in *COL1A1* (Figure 7A), increase in *COL2A1* (Figure 7B), increase in *ACAN* (Figure 7C) and decrease in *COL10A1* (Figure 7D) in HAMA-containing gels. Whilst some slight variability existed within HAMA-free and HAMA-containing gels, no significant difference was detected with the presence of HAP.

### 3.7. Immunofluorescence

**Figure 8:**
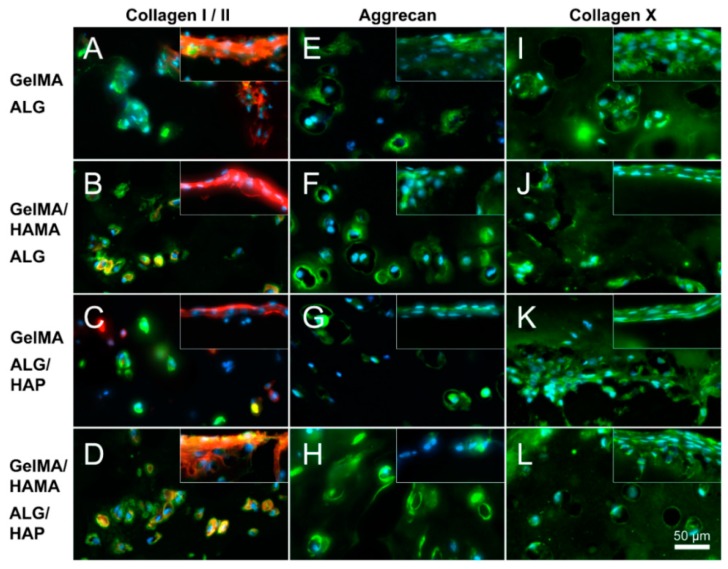
Immunofluorescence images illustrating deep and surface sections. Staining of collagen I (red), collagen II (green), aggrecan (green) and collagen X (green) was performed on constructs from all groups on day 28 (as indicated; DAPI nuclei staining in light blue); (**A**,**E**,**I**): GelMA-ALG; (**B**,**F**,**J**): GelMA/HAMA-ALG; (**C**,**G**,**K**): GelMA-ALG/HAP and (**D**,**H**,**L**): GelMA/HAMA-ALG/HAP. Images were taken from the deep hydrogel zone near to the ALG or ALG/HAP scaffold, with surface images inset; scale bar 50 µm.

Immunofluorescence was conducted on sections to corroborate gene expression data on an ECM-protein level. Staining was done for collagen I and II (I red, II green, Figure 8A–D), aggrecan (Figure 8E–H) and collagen X (Figure 8I–L) performed on all groups (top row: GelMA-ALG, second row: GelMA/HAMA-ALG, third row: GelMA-ALG/HAP, bottom row: GelMA/HAMA-ALG/HAP). Chondrocyte spreading and high collagen I expression was clearly observable at the surface of the gels compared with the interior. Within the center of the gels a chondrogenic response was confirmed through a more rounded chondrocyte morphology and collagen II staining. Aggrecan staining was found throughout the matrix of all groups as well as at the surface. Collagen X appeared present throughout the ECM, with strongest staining apparent at the HAP interface (as seen in the lowest area of panel K). Images were taken of the deep portion of hydrogels at the periphery of the scaffold meshes, with surface images inset. Overall a chondrogenic effect of HAMA was observed, with a very limited influence of HAP. Isotype negative controls in the form of mouse IgG were used to assess non-specific staining (data not shown).

## 4. Discussion

HAP-containing (gelMA-ALG/HAP, gelMA/HAMA-ALG/HAP) and HAP-free (gelMA-ALG, gelMA/HAMA-ALG) scaffolds were produced through 3D plotting of alginate or alginate/hydroxyapatite paste and photocrosslinked with hydrogels to form hybrid constructs for evaluation of preliminary osteochondral regeneration. Whilst hydrogels widely appear to give the most desired matrix in terms of hypoxia, high fluid content, ease of diffusivity and tailorable mechanical properties, there is no clear consensus on what type of material is most desirable for osteochondral regeneration [13,25,26,27,28,29]. Herein we presented a hybrid construct containing both chondro- and osteo-conductive materials. However, it is a preliminary design which does not afford the clear material-driven effects we may observe when using homogeneous structures. Khanarian and colleagues present such a construct, showing that in a large volume scaffold with a homogenous composition, the potential of hydroxyapatite as a ZCC material is clearly shown [27]. In this study, alginate was used as the hydrogel component of the ceramic/hydrogel scaffold, although unlike in our study, the construct was not formed using 3D plotting. Alginate has previously been shown to be both supportive of chondrocytes in culture [30,31,32], and valid as a tissue engineering vessel [33,34,35]. It also has desirable viscous properties for 3D plotting and ionic crosslinking capabilities that allow for a rapid sol-gel transition. Furthermore, alginate has been shown to be biocompatible and biodegradable, supportive of native chondrocyte morphology and matrix deposition, as well as promoting bone healing in certain cases [36,37]; all of which led to our selection of alginate as a component of our ceramic paste. Our team has also recently published work involving a somewhat similar concept to the plotting mechanism presented herein, finding that 3D plotting of gelatin/alginate with or without HAP was an efficacious scaffold system, supporting bone marrow stromal cells over 21 days *in vitro* [38].

Interestingly, the limited chondrocyte hypertrophy we observed (as evidenced by *COL10A1* expression and immunofluorescent staining) was similar for Khanarian *et al.* [27], who reported that the exclusive use of deep zone chondrocytes gave a much better result in terms of hypertrophy and mechanical properties when compared with full thickness cells. It may be inferred that subdividing constructs in the future to isolate cell populations may be advantageous. For instance, the calcified layer of the scaffold may be printed with cells included within the paste, and/or a small layer of hydrogel containing deep zone chondrocytes may be photocrosslinked onto the mesh, followed by a layer of hydrogel containing superficial or full thickness chondrocytes for the remainder of the construct. Alternately, the printing of the entire construct may be possible if appropriate material optimizations are performed to ensure structural fidelity. Whilst the material choice is supported by literary evidence and conducive to chondrogenesis, there is a limited effect on calcification that indicates the scaffold system requires further development and characterization.

Use of layered constructs has been explored extensively in the literature, with three main methods used: (1) seeding of chondrocytes or neocartilage tissue directly onto a bone scaffold; (2) “assembling” a bilayered scaffold from two components; and (3) forming an “integrated” bilayered scaffold [39]. Our approach falls into category 3, and is also supported in principle and material choice (alginate) by a study focused on 3D plotting of layered scaffolds, where preliminary outcomes show chondrogenesis both *in vitro* and *in vivo* [13]. Data related to enhanced mechanical properties also reinforce the relevance of this work, with Hollenstein *et al.*, showing improved bone-hydrogel interfacing when a ZCC was included in constructs [25]. Whilst all the aforementioned studies delve into innovations within this field, the fact remains that further study is required in order to completely construct a relevant picture of what is required for successful osteochondral regeneration through the implementation of scaffold technologies. Namely, it is difficult to determine which chondrogenic and osteogenic components are most efficacious for the regeneration of a single component. In a combined environment, it is also difficult to determine whether their applicability to the regeneration varies upon combination or is affected by a biphasic system. In our approach we used a chondrogenic medium containing transforming growth factor (TGF)-β3. It has been shown that TGF-β3 is involved in both chondrogenic [40] and osteogenic [41,42] differentiation, and hence it was considered as conducive to development in both areas of our construct. Future studies may benefit from an alternate culture approach however, as TGF-β3 has been shown to decrease scar tissue formation through the attenuation of collagen type I synthesis; however these effects are largely demonstrated on skin lesions postoperatively and hence in fibroblastic cells [43,44,45]. Its effects on collagen type X synthesis are currently undefined and conversely to the above, it has also been observed to stimulate transcription of the *COL1A2* gene [46]. Hence the level to which it may be influential on the development of the ZCC are yet undetermined and require further testing.

Overall, in this study we presented a preliminary structure consisting of a mesh scaffold combined with a hydrogel polymer, and show that the combination yields a greater increase in mechanical properties than that of a control scaffold (ALG), and that the use of the ALG/HAP scaffolds does not negatively influence chondrogenic response of chondrocytes *in vitro.* Limited cytotoxicity was observed on day 1 from either of the two scaffold types or the gels, as confirmed by fluorescent staining and subsequent image quantification. While viability was not assessed at later time-points, increases in DNA and GAG content indicated that cells remained viable throughout the entire 28 day culture period. Elastic modulus increased considerably throughout the culture period, with HAMA significantly increasing modulus in ALG groups and the use of ALG/HAP scaffolds increasing modulus relative to the ALG groups. It may be reasoned that the addition of hyaluronic acid within the hydrogel positively influenced both the cell activity throughout the culture period, as well as providing an additional material that is able to crosslink to both its own and to the gelMA network.

There was an increased trend in modulus with ALG/HAP, which may be due to the significantly higher density in the scaffold. Scaffold strands were printed with the same diameter nozzle in both cases (ALG and ALG/HAP) and hence with the HAP groups a lesser proportion of water was present in each strand. The HAP content was in fact 42%, and hence the concentration of alginate in water would also be higher in the ALG/HAP scaffold strands compared with the ALG alone. However, simultaneously the alginate network integrity may be disturbed by the presence of HAP particles within the construct strands, and any benefit to the increased concentration of ALG within the strand may be mitigated. Finally, if indeed the ALG/HAP scaffold matrix was stiffer than ALG, the increase in overall construct modulus may be caused by a lesser transverse expansion of the scaffolds under load. This hypothesis is supported by Khanarian *et al.* [27], who demonstrate an increase in modulus of scaffold constructs made entirely from alginate and hydroxyapatite, and hence it may be concluded that the presence of the HAP does in fact influence the stiffness of the construct strands. The mechanical properties obtained herein are over an order of magnitude lower than that of native cartilage tissue, however the mimicry of native values may be not required. The construct must of course be able to withstand the mechanical environment of the joint, but with appropriate stimulus (driven through the neotissue matrix) the matrix deposition of chondrocytes may be encouraged. As this model was cultured in a static environment, the values of modulus are not representative of what would be attainable under compressive loading during culture. This was done however to simplify the process and allow the examination of parameters in a less complex system. It is unclear what mechanical properties are ideal for a tissue engineered cartilage construct, however it is the belief of the authors that this will become clearer as biomaterials research progresses.

Examination of GAG and DNA content within the scaffolds showed consistent DNA levels between groups, and a comparable yet significantly different level of GAG secreted between the gelMA and the gelMA/HAMA groups. In all cases HAMA increased the level of GAGs produced, yet the varying of scaffold type from ALG to ALG/HAP failed to have an effect on this metric. It may be that the localization of the scaffolds to the greatest depth of the gels along with their physiologically relevant thickness reduces any potential effects they may have on the majority of the chondrocytes within the gel phase. However, it is clear that inclusion of a prominent glycosaminoglycan such as HA within the gel has a positive effect on chondrogenic differentiation, whilst it is unknown what effect methacrylation of this compound has on its cellular interactions. This data confirms our research group’s previous findings, where HAMA was found to be supportive of chondrogenic differentiation [16]. As all GAG data were corrected using cell-free constructs, the influence of HAMA on the GAG assay was negated.

PCR data showed a much higher expression of *COL1A1* and *COL10A1* in the HAMA-free gels, indicating a fibrocartilaginous phenotype, whilst *COL2A1* and *ACAN* expression further supported this conclusion. Higher levels of collagen I and collagen X are indicative of dedifferentiated chondrocytes, with elevated levels observed in gelMA-only gels throughout the literature [16,47]. This effect is namely due to the high number of attachment (RGD peptide) sites present on gelatin that strongly promote cell attachment and hence spreading, along with the fact that the polymer is a derivative of largely collagen type I.

Higher expression levels of chondrogenically relevant genes such as *COL2A1* and *ACAN* were observed in HAMA-containing gels compared against HAMA-free groups. There was no significant effect of ALG/HAP scaffolds on *COL10A1* expression, although a slight trend was noticeable. It is possible that downregulation of *COL1A1* and *COL10A1* in hydrogels containing HAMA was caused by activation of CD44, as in low concentrations hyaluronic acid has been shown to promote chondrogenesis through this pathway [48,49]. Immunofluorescence examining protein levels in the matrix indicated similar relationships to PCR, with higher collagen I staining found in HAMA-free gels and stronger collagen II and aggrecan staining observable in HAMA-containing gels. Collagen X staining appeared relatively even throughout the ECM, with gelMA-ALG/HAP showing staining surrounding the lowest region of the scaffold which was the area of interest in this case (Figure 8K). The levels in all groups did not vary enough to show conclusive effects, however trends appeared to support PCR findings and hence are also explainable and in support of the previously listed reasoning. In all cases isotype negative controls consisted of mouse IgG and verified no non-specific staining of matrix occurred.

In line with previous results [25,27,50], we found a slight increase in modulus after culture of ALG/HAP scaffolds, with comparable trends in cell free conditions. On the other hand, whilst others have shown that inclusion of bioactive glass increases GAG in hydrogels [28], we found ALG/HAP to have a limited effect in our system. As the ALG/HAP scaffolds were intact at the time of crosslinking and appeared to remain intact at day 28 of culture, it may be postulated that limited diffusion of HAP occurred throughout the culture period. It may thus be inferred that culture in a system such as a compression bioreactor may further accentuate the observed trends and bring them to a significant and notable level. With appropriate structural modifications, perfusion bioreactors may also be suitable for this application [36]. Overall we showed that the combination of gelMA and HAMA with a HAP/ALG scaffold system is viable for cells, maintains positive chondrogenesis and provides a platform for the exploration of innovation into such scaffold technologies.

## 5. Conclusions

Scaffolds comprising human articular chondrocyte-containing hydrogels (GelMA or GelMA/HAMA) crosslinked through 3D printed paste meshes (alginate or alginate/HAP) were cultured for 28 days under chondrogenic conditions *in vitro*. No cytotoxicity was observed from scaffold components. Incorporation of HAMA into hydrogels improved chondrogenesis. The use of ALG/HAP *versus* ALG did not encourage the formation of a ZCC, however changes in scaffold design, such as incorporation of a larger amount of HAP, or use of bioreactor systems may be used in the future to expand on this system.
